# A retrospective audit of whether patients undergoing myocardial perfusion scintigraphy (MPS) are suitable for Stress cardiac magnetic resonance imaging (CMR)

**DOI:** 10.1186/1532-429X-15-S1-E72

**Published:** 2013-01-30

**Authors:** Mark Peterzan, Adam Timmis, Daniel Moffat, Ceri Davies, Saidi A Mohiddin, Anthony Mathur, Charles Knight, Mark A Westwood, Neha Sekhri

**Affiliations:** 1 London Chest Hospital, London, UK

## Background

The recent NICE guidelines (UK) have recommended functional testing in patients with intermediate probability of coronary artery disease. The choice of functional tests (Stress CMR, MPS, stress echo) is largely dependent on local expertise and available resources. Our institute is a leading UK CMR (1.5T) centre performing >3500 adenosine stress CMRs a year. Recent studies like CE-MARC have further confirmed the non-inferiority of stress CMR. Our aim was to identify the number and reasons why myocardial perfusion scans were being performed, despite an established stress CMR service. Keeping in mind the local tariffs, an MPS scan costs £198.60 more than a stress CMR scan, notwithstanding the risk of exposure to iononizing radiation.

## Methods

All patients undergoing MPS from 1 April 2011 to 31 March 2012 were identified. Individual patient data were extracted from their electronic health records to identify the indication for scanning, the history of coronary disease, the grade and specialty of the referring clinician, any previous cardiac imaging and any potential contraindications to CMR (GFR<30, non CMR safe devices, claustrophobia, high body mass index). This study was undertaken as part of clinical evaluation of service.

## Results

A total of 322 patients underwent MPS scans during the study period.17 patients whose scans were for research purposes were excluded. At the time of the MPS study, 14 patients had previously undergone CMR, 26 cardiac CT angiogram and 156 invasive angiography. Indications for MPS in the 305 study patients were: shortness of breath (n=51), assessment of angina (n=127), atypical chest pain (n=126), non-specific symptoms (n=57) and pre-assessment prior to surgery (n=23). 58% (176/305) patients had no a priori contraindications to CMR. Contraindications recorded were: MR unsafe pacemaker (n=17), ICD/ CRT-D (n=19), GFR <30 ml/min (n=30), claustrophobia (n=43), increased body mass >120 kg (n=9), other metal implant (n=3) and data was unavailable for 10 patients. Some patients had >1 contraindication. Of 176 (58%) patients without contraindication to CMR, 103 had no previously documented CAD and 97 had had no previous cardiac imaging. 88% (155/176) had a cardiac source of referral (rapid access chest pain clinics n=76 and consultant cardiology clinic n=79), 6% (n=10/176) by the non-cardiologists and it was unclear in 6% (n=11/176) patients.

## Conclusions

Our audit has identified that in a centre with well-established stress CMR service, where we have shown significant safety and cost benefits favouring CMR perfusion, eligible patients are still being referred for MPS scan and the majority from cardiac services. Further work to understand the reasons why MPS were ordered, may be key to making considerable cost and safety gains.

## Funding

None.

